# Supplementary material for: Response to immune checkpoint blockade improved in pre-clinical model of breast cancer after bariatric surgery
Source: eLife. 2022 Jul 1;11:e79143. doi: 10.7554/eLife.79143 (PMC9342954; doi:10.7554/eLife.79143)
Supplement: Supplementary file 1. — (a) Multiple comparisons of body weight after surgery over time. *p<0.05, **p<0.01, ***p<0.001, and ****p<0.0001. Two-way ANOVA with Fisher’s LSD test. Low fat diet (LFD), high fat diet (HFD), vertical sleeve gastrectomy (VSG), and weight-matched (WM). (b) Multiple comparisons of tumor volume over time. *P<0.05, **P<0.01, ***P<0.001, ****P<0.0001. Two-Way ANOVA with Fisher’s LSD test. LFD, HFD, VSG, and WM. (c) Conserved differentially expressed genes in subcutaneous adipose/mammary fat pad in obese and bariatric surgery patients and mice. [file elife-79143-supp1.docx]

**Supplemental File 1a. Multiple comparisons of body weight after surgery over time.**

*p<0.05, **p<0.01, ***p<0.001, ****p<0.0001. Two-Way ANOVA with Fisher’s LSD test.

Low fat diet (LFD), High fat diet (HFD), Vertical sleeve gastrectomy (VSG), Weight-Matched (WM)

|  | Weeks after surgery | | | | | | | | | | |
| --- | --- | --- | --- | --- | --- | --- | --- | --- | --- | --- | --- |
|  | 0 | 0.5 | 1 | 1.5 | 2 | 2.5 | 3 | 3.5 | 4 | 4.5 | 5 |
| LFD-Sham vs. HFD-Sham | **** | **** | **** | **** | **** | **** | **** | **** | **** | **** | **** |
| LFD-Sham vs. HFD-VSG | **** | **** | **** | **** | *** | *** | ** | *** | ** | *** | **** |
| LFD-Sham vs. WM-Sham | **** | **** | **** | **** | *** | ** | ** | ** | * | * | ** |
| HFD-Sham vs. HFD-VSG | ns | ns | *** | **** | **** | **** | **** | **** | **** | **** | **** |
| HFD-Sham vs. WM-Sham | ns | ns | * | ** | **** | **** | **** | **** | **** | **** | **** |
| HFD-VSG vs. WM-Sham | ns | ns | ns | ns | ns | ns | ns | ns | ns | ns | ns |

**Supplemental File 1b.** **Multiple comparisons of tumor volume over time.**

*p<0.05, **p<0.01, ***p<0.001, ****p<0.0001. Two-Way ANOVA with Fisher’s LSD test.

Low fat diet (LFD), High fat diet (HFD), Vertical sleeve gastrectomy (VSG), Weight-Matched (WM)

|  | Weeks after tumor implantation | | | | |
| --- | --- | --- | --- | --- | --- |
|  | 1 | 1.5 | 2 | 2.5 | 3 |
| LFD-Sham vs. HFD-Sham | *** | ** | **** | *** | **** |
| LFD-Sham vs. HFD-VSG | * | ns | ns | * | ** |
| LFD-Sham vs. WM-Sham | ns | ns | ns | ns | ns |
| HFD-Sham vs. HFD-VSG | ns | * | * | ns | * |
| HFD-Sham vs. WM-Sham | ** | ** | **** | *** | **** |
| HFD-VSG vs. WM-Sham | ns | ns | ns | ** | ** |

**Supplemental File 1c. Conserved differentially expressed genes in subcutaneous adipose/mammary fat pad in obese and bariatric surgery patients and mice.**

| **Gene symbol** |
| --- |
| Ret |
| Ddah1 |
| Hp |
| Lpgat1 |
| Nek6 |
| Ankrd50 |
| Sparc |
| Tuft1 |
| Rab20 |
| Chka |
| Dgki |
| Lep |
| Tgm1 |
| Itga1 |
| Tmem125 |
| Cd200 |
| Slc7a4 |
| Msc |
| Usf2 |
| Ephb3 |
| Cntn2 |
| Lgals7 |
| Mast4 |
| Tusc1 |
| Aldoc |
| Klhl5 |
| Arhgap20 |
| Setd7 |
| Thoc2 |
| Nap1l1 |
| Nkiras1 |
| Cmtm8 |
| Serpinf1 |
| Psme4 |
| Col4a1 |
| Clca2 |
| Nrp2 |
| Ficd |
| Kctd10 |
| Rtn4rl1 |
| Eif4b |
| Vgll3 |
| Slc15a4 |
| Slc35g1 |
| Pde8a |
| Mid1 |
| Tarsl2 |
| Sema3c |
| Pcdh7 |
| Vps13a |
| Amn1 |
| Ido1 |
| Npr3 |
| Srsf4 |
