# Supplementary material for: Response to immune checkpoint blockade improved in pre-clinical model of breast cancer after bariatric surgery
Source: eLife. 2022 Jul 1;11:e79143. doi: 10.7554/eLife.79143 (PMC9342954; doi:10.7554/eLife.79143)
Supplement: Supplementary file 2. — Total cells from tumor or tumor adjacent mammary fat pad (including tumor draining lymph node, TdLN) were gated by plotting forward scatter area versus side scatter area, single cells by plotting side scatter height versus side scatter area, live cells by plotting side scatter area versus Ghost viability dye, and immune cells by plotting CD45 versus Ghost viability dye. T cells were gated as follows: CD3+ T cells (CD3+) and CD8 + T cells (CD3+ and CD8+). Mean fluorescent intensity (MFI) of PD-1 was measured in CD3+ PD-1+ cells. Monocytic myeloid derived suppressor cells (M-MDSC) are gated as CD11b+, Ly6Chigh, and Ly6G−. Macrophages are gated as CD11b+ and F480+. Non-immune cells were gated as CD45−, PD-L1+, and MFI for PD-L1. [file elife-79143-supp2.docx]

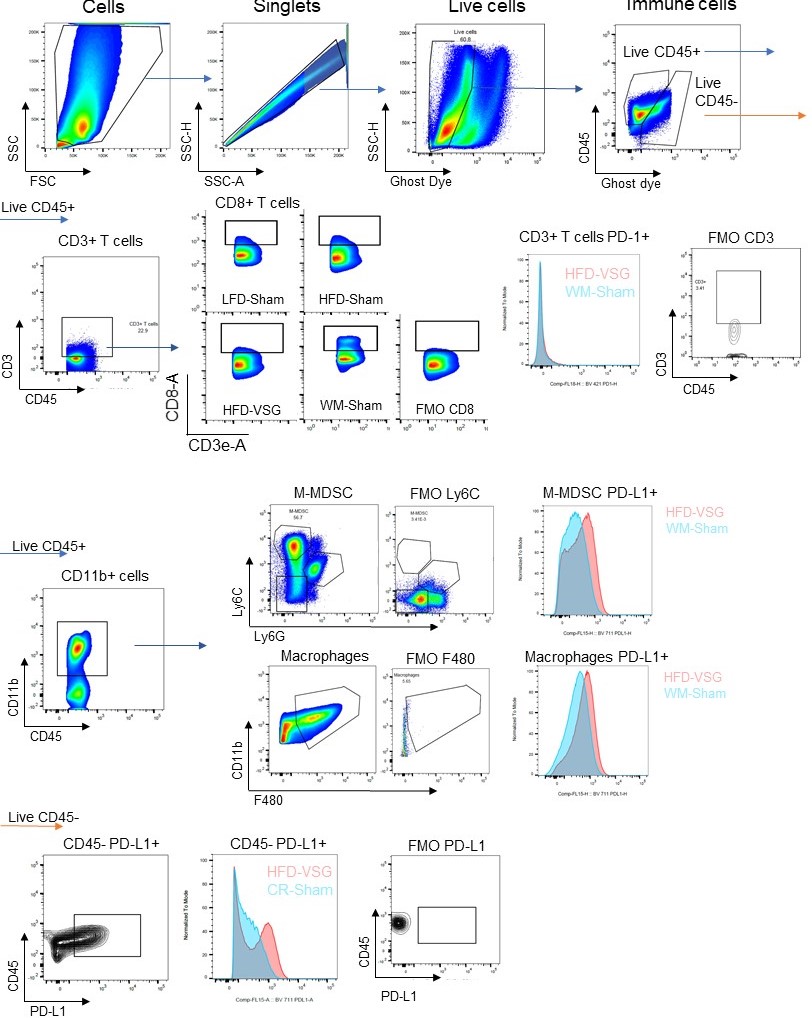
**Supplemental File 2. Gating schema for flow cytometric analysis of immune cells in tumor single cell suspensions.** Total cells from tumor or tumor adjacent mammary fat pad (including tumor draining lymph node, TdLN) were gated by plotting forward scatter area versus side scatter area, single cells by plotting side scatter height versus side scatter area, live cells by plotting side scatter area versus Ghost viability dye, and immune cells by plotting CD45 versus Ghost viability dye. T-cells were gated as follows: CD3+ T cells (CD3+), and CD8+ T cells (CD3+, CD8+). MFI of PD-1 was measured in CD3+ PD-1+ cells. Monocytic myeloid derived suppressor cells (M-MDSC) are gated as CD11b+, Ly6C^high^, Ly6G-. Macrophages are gated as CD11b+, F480+. Non-immune cells were gated as CD45-, PD-L1+, and MFI for PD-L1.
